# Problem drinking and comorbidity with mental ill health: a cross-sectional study among healthcare workers in Sweden

**DOI:** 10.1093/alcalc/agaf016

**Published:** 2025-04-17

**Authors:** Josefina Peláez-Zuberbuhler, Emelie Thern, Håvard R Karlsen, Siw Tone Innstrand, Marit Christensen, Bodil J Landstad, Devy L Elling, Malin Sjöström, Emma Brulin

**Affiliations:** Unit of Occupational Medicine, Institute of Environmental Medicine, Karolinska Institutet, Solnavägen 4, 113 65 Stockholm, Sweden; Department of Psychology, Norwegian University of Science and Technology, 7049 Trondheim, Norway; Department of Leadership and Organization, Kristiania University College, Prinsens gate 7-9, 0107 Oslo, Norway; Unit of Occupational Medicine, Institute of Environmental Medicine, Karolinska Institutet, Solnavägen 4, 113 65 Stockholm, Sweden; Department of Psychology, Norwegian University of Science and Technology, 7049 Trondheim, Norway; Department of Psychology, Norwegian University of Science and Technology, 7049 Trondheim, Norway; Department of Psychology, Norwegian University of Science and Technology, 7049 Trondheim, Norway; Faculty of Human Sciences, Mid Sweden University, 831 25 Östersund, Sweden; Unit of Research, Education and Development, Östersund Hospital, 831 50 Östersund, Sweden; Unit of Occupational Medicine, Institute of Environmental Medicine, Karolinska Institutet, Solnavägen 4, 113 65 Stockholm, Sweden; Department of Public Health and Clinical Medicine, Umeå University, 901 87 Umeå, Sweden; Unit of Occupational Medicine, Institute of Environmental Medicine, Karolinska Institutet, Solnavägen 4, 113 65 Stockholm, Sweden

**Keywords:** problem drinking, healthcare, burnout, depression

## Abstract

**Aims:**

Problem drinking in healthcare workers (HCWs) is highly relevant to study as it could result in personal suffering, as well as inefficiencies in health service delivery. This study aims to investigate the prevalence of nondrinking, drinking, and problem drinking and to investigate the comorbidity between drinking alcohol and mental illness (burnout and depression) among HCWs in Sweden.

**Methods:**

This cross-sectional study draws on the 2022 Longitudinal Occupational Health survey in Healthcare Sweden of physicians, nurses, and nurse assistants in Sweden (*N* = 5966). Measures include levels of alcohol use assessed by the Cut, Annoyed, Guilty, and Eye Opener questionnaire, the 12-item Burnout Assessment Tool, and the Symptom CheckList–Core Depression. Multinomial Logistic regressions were used to investigate the likelihood of reporting nondrinking and problem drinking compared to drinking.

**Results:**

The prevalence of problem drinking among Swedish HCWs was 3.7%. Only sex differences were observed for those with a problem drinking, with male nurses and nurse assistants being more likely to report problem drinking. Comorbidity was found between problem drinking and depression but not between problem drinking and burnout.

**Conclusions:**

This study demonstrated that ~3.7% of Swedish HCWs had problem drinking and that those also had a higher likelihood of reporting depression but not burnout. Results contribute to new knowledge about the use of alcohol and comorbidities with depression and burnout among HCWs in Sweden. Findings could benefit employers in implementing preventive and tailored strategies to preserve the psychosocial well-being of HCWs.

## Teaser

This study examined problem drinking and mental health among healthcare workers (HCWs) in Sweden. Problem drinking was found in 3.7% of HCWs, with male nurses and nurse assistants at higher risk. Comorbidity was found between problem drinking and depression but not between problem drinking and burnout.

## Introduction

From a societal perspective, problem drinking, i.e. individuals who consume large amounts of alcohol or occasionally face problems due to drinking but do not have a background of severe physical dependence on alcohol (Walitzer and Connors 1999) by healthcare workers (HCWs), is highly relevant as it could result in poor health and inefficiencies in health service delivery (de Lange *et al*. 2024). The high risk of alcohol consumption can compromise HCWs’ practice of medicine, decreasing their performance and influencing their behaviours regarding the quality of care and safety of patients (Searby *et al*. 2024). For the individual HCW, problem drinking can lead to adverse work-related outcomes and behaviours, such as the risk of being injured in the workplace (Borrelli *et al*. 2023), mental health problems (Tao *et al*. 2023), and increased sickness absence, all of which can also pose subsequent adverse consequences for the quality of care and patient safety (de Lange *et al*. 2024).

Studies show that HCWs working in demanding work environments, which many do (Gynning *et al*. 2024), may use alcohol as a coping strategy (Ross *et al*. 2018; Halsall *et al*. 2023). Recent studies show that the pooled prevalence of harmful alcohol use among HCWs was 3.2% (Halsall *et al*. 2023), although substantial variations exist across countries (Mo *et al*. 2022). For instance, reported prevalence rates include 1.2% among French physicians (Thiebaud *et al*. 2021), 2.3% among Australian physicians (Wijeratne *et al*. 2021), 5.5% among Australian nurses (Searby *et al*. 2023), and 0.7% among American physicians (Foli *et al*. 2021). A recent Swedish study shows that the prevalence of problem drinking in the health and social care sector was 4.7% in 2020, lower than the prevalence among all included workers, which was 6.6% (Thern *et al*. 2024). Another Swedish study conducted prior to the COVID-19 pandemic shows that HCWs had a relatively low rate of hazardous alcohol consumption, i.e. alcohol consumption that confers risk but has not yet reached the level of problem drinking, compared to other sectors (Tareq *et al*. 2024).

A recent review shows that the prevalence of alcohol use disorder varies between physicians and other HCWs, including nurses, depending on the type of measurement, indicating the need to further compare the prevalence of problem drinking across different healthcare professions (Waithera *et al*. 2024). Various international studies suggest that male physicians are more likely to report a alcohol misuse compared to female physicians (Rosta and Aasland 2013; Sørensen *et al*. 2016; Mo *et al*. 2022), although a recent systematic review showed that sex did not predict the occurrence of hazardous alcohol use in HCWs (Halsall *et al*. 2023). Other studies revealed a higher percentage of risky consumption among female HCWs (Oreskovich *et al*. 2015; Cedrone *et al*. 2022). Problem drinking in HCWs has also been associated with increased years of service and hours worked (Schluter *et al*. 2012). Lastly, parental responsibilities have also been associated with the use of alcohol among physicians and nurses, and not having children increases the likelihood of drinking problems (Thiebaud *et al*. 2021; Beiter *et al*. 2022). Despite clear evidence of the risks of consuming alcohol, the variation in prevalence across countries, and the variations across care professions and sociodemographic factors, there is still a lack of prevalence studies describing Swedish HCWs’ alcohol use and harmful use (Halsall *et al*. 2023).

There is extensive literature that supports that comorbidity between problem drinking and mental ill health exists, i.e. a simultaneous presence of two or more health conditions. For instance, review studies show that alcohol use disorders were higher among those with any common mental disorders compared to those without (Jane-Llopis and Matytsina 2006; Waithera *et al*. 2024). However, findings on this relationship remain inconsistent; for instance, a South African study found no significant univariate correlation between alcohol use disorder and reported depression and anxiety (Mc Magh *et al*. 2023). Considering the high prevalence of mental ill health, i.e. depression and burnout, among HCWs (Brulin *et al*. 2023), comorbidity should be further investigated.

This study aims to investigate the prevalence of self-reported nondrinking, drinking, and problem drinking among HCWs in Sweden and to investigate the comorbidity between problem drinking and mental illness (burnout and depression). Knowledge about the extent of problem drinking, at-risk groups, and comorbidity is needed to design effective interventions to prevent problem drinking among HCWs (Oreskovich *et al*. 2015). Addressing problem drinking within this professional group may contribute to reducing sickness absence, enhancing job performance, and improving overall patient safety. Findings will also be critical for informing workplace policies aimed at promoting HCWs’ well-being and ensuring the provision of safe and high-quality healthcare.

## Material and Method

### Sample and data collection

This cross-sectional study draws on data from the 2022 Longitudinal Occupational Health survey in HealthCare Sweden (LOHHCS). Using stratified random sampling with six geographical strata, a representative sample of 7908 physicians, 7790 nurses, and 7967 nurse assistants were drawn from the Swedish National Occupational based on Swedish Standard Classification of Occupations (SSYK) codes and the National Educational Registers based on degree. Statistics Sweden was responsible for sampling, questionnaire distribution, and data collection.

Postal invitations to participate in the study, including personal log-in information for a web-based survey, were sent from Statistics Sweden to the study sample. Three reminders were sent by postal mail. A paper version of the survey was included in the second reminder. The response rates were 34.3% (*n* = 2712) for physicians, 37.3% (*n* = 2903) for nurses, and 26.7% (*n* = 2043) for nurse assistants. Statistic Sweden analysed missing data by comparing the characteristics of the responders to the sample and the full population using various national registers and found no systematic differences. According to retirement age, this study restricted the sample to those >68 years. This resulted in an analytical sample of 7589 HCWs (2143 physicians, 2903 nurses, and 2043 nurse assistants).

The Swedish Ethical Review Authority approved the study (2020-06613; 2021-05574-02; 2022-00310-02). Participants were informed about the study’s voluntary and anonymous nature and were not compensated for participating.

### Measurements

‘Problem drinking’ was assessed using a modified version of the Cut, Annoyed, Guilty, and Eye Opener (CAGE) questionnaire (Ewing 1984). Problem drinking refers to alcohol consumption that has resulted in problems, detectable at both clinical and subclinical levels by the CAGE questionnaire (Ewing 1984). The respondents were first asked, ‘Do you regularly drink alcohol?’ with a five-point Likert-type scale ranging from ‘never’ (1) to ‘every day’ (5). Those who responded two to five continued to answer the CAGE, which contains four items pertaining to their lifetime drinking experience (i.e. ‘Have you ever felt you should cut down on your drinking?’). Each item has a 0 (no) and 1 (yes) score. A CAGE score <2 indicated drinking, and ≥2 indicated problem drinking. We then created a variable with three categories: nondrinking, drinking (i.e. use of alcohol that is not harmful), and problem drinking.

Self-perceived ‘burnout’ was measured using the Burnout Assessment Tool 12 (BAT-12) (Schaufeli *et al*. 2020). Participants were asked to rate statements (e.g. ‘When I am at work, I feel mentally exhausted’) using a five-point Likert-type scale ranging from ‘never’ (1) to ‘always’ (5). Cronbach’s alpha for all 12 items was 0.90, indicating high internal consistency. A grand mean score was computed, and a cut-off score of ≥2.96 indicated suffering from burnout complaints (Schaufeli *et al*. 2023) (heron burnout).

Self-rated ‘depression’ was measured using the Symptom Check List–Core Depression 6 (SCL-CD6) (Magnusson Hanson *et al*. 2014). The scale consists of six items regarding symptoms of depression during the last 7 days (i.e. feeling blue/sad, no interest in things, low energy, everything is an effort, worrying too much, and blaming oneself). All the items were rated on a Likert scale ranging from ‘Not at all’ (0) to ‘Extremely’ (4). Cronbach’s alpha for all six items was 0.91, indicating high internal consistency. The sum score was computed for the overall scale, and a cut-off score was set at 17 points, indicating that individuals with scores of ≥17 most probably have major depression (Magnusson Hanson *et al*. 2014).

#### Confounders

Based on previous research (Waithera *et al*. 2024), sex, age, years of work experience, and family constellation were used as potential confounders. Data on sex (men and women) and age were retrieved by Statistics Sweden from the Swedish population register. Years of work experience (<5, 5–10, 10–15, >15), having a partner (yes/no), and having children (yes/no), were self-reported.

### Data analysis

Descriptive statistics were used to identify the prevalence of nondrinking, drinking, and problem drinking for the total sample across each demographic variable (profession, sex, age, work experience, partner, children) and mental ill health (burnout and depression). Analysis of variance (ANOVA), with a *post hoc* test, was used to assess potential significant differences in the mean level of mental ill health between the three groups nondrinking, drinking, and problem drinking.

Next, multinomial logistic regressions were conducted to test the likelihood of reporting nondrinking, drinking (base category), and problem drinking across professions and demographics. First, simple effect models (crude model) were conducted for each variable. Next, an adjusted model (Model 1) was carried out, including all demographic variables. To test for comorbidity, two models (Models 2 and 3) additionally added burnout and depression, respectively, due to a high correlation between the two variables (*r* = 0.76, *P* < .001). Lastly, stratified analyses for Models 2 and 3 per profession were conducted. Results were presented with odds ratios (ORs) and 95% confidence intervals (CIs). Statistical analyses were performed using the IBM Statistical Package for Social Sciences (SPSS, version 29.0).

## Results

### Study sample and descriptive statistics

The study sample comprised 34.7% physicians, 38.1% nurses, and 27.2% nurse assistants (Table 1). The mean age for the study sample was 46.8 (SD 11.9), and most respondents were women. Half of the sample had >15 years of professional experience (50.6). While almost all had a partner (84.0%), only about half (49.3%) had children at home.

**Table 1:** Descriptive statistics of nondrinking, drinking, and problem drinking

	Total	Nondrinking	Drinking	Problem drinking
Total *n* (%)	7392	1448 (19.6)	5672 (76.7)	272 (3.7)
Profession *n* (%)				
Physicians	2588 (34.7)	394 (15.3)	2085 (81.0)	94 (3.7)
Nurses	2844 (38.1)	490 (17.4)	2218 (78.6)	113 (4.0)
Nurse assistants	2030 (27.2)	564 (28.2)	1369 (68.5)	65 (3.3)
Sex *n* (%)				
Male	1518 (20.5)	241 (15.9)	1204 (79.5)	73 (4.8)
Female	5874 (79.5)	1207 (20.5)	4468 (76.1)	199 (3.4)
Work experience (years) *n* (%)				
<5	1150 (15.6)	218 (19.0)	893 (77.7)	39 (3.4)
5–10	1280 (17.4)	290 (22.7)	948 (74.1)	42 (3.3)
10–15	1211 (16.4)	285 (23.5)	876 (72.3)	50 (4.1)
>15	3728 (50.6)	653 (17.5)	2934 (78.7)	141 (3.8)
Partner *n* (%)				
Yes	6194 (84.0)	1136 (18.3)	4830 (78.0)	228 (3.7)
No	1178 (16.0)	308 (26.1)	826 (70.1)	44 (3.7)
Children living in the home *n* (%)				
Yes	3562 (49.3)	755 (21.2)	2668 (74.9)	139 (3.9)
No	3659 (50.6)	644 (17.6)	2889 (79.0)	126 (3.4)
Age *m* (StD)	46.8 (11.9)	46.4 (11.6)	46.8 (12.0)	47.63 (11.2)
Burnout *n* (%)	501 (6.7)	145 (28.9)	332 (66.3)	24 (4.8)
Depression *n* (%)	462 (6.3)	126 (27.3)	299 (64.7)	37 (8.0)

### Prevalence and comorbidity

A fifth of the HCWs had not used alcohol in the last 12 months (nondrinkers), and 3.7% reported a problem drinking (Table 1). Problem drinking was more common among nurses (4.0%), males (4.8%), and those with 10–15 years of experience (3.8%) and children living at home (3.9%).

The ANOVA (Table 2) showed that among HCWs reporting problem drinking, the mean levels of depression (*M* = 8.50) and burnout (*M* = 2.10) were significantly higher compared to those drinking and not drinking alcohol, while the mean levels among HCWs reporting drinking were the lowest for both depression (*M* = 5.83) and burnout (*M* = 1.89).

**Table 2:** Mean comparisons for mental ill health for nondrinking, drinking, and problem drinking with *post hoc* Tukey test

	Nondrinking	Drinking	Problem drinking	P-value for difference between Nondrinking and drinking	P-value for difference between nondrinking and problem drinking
Burnout (1–5)	1.99 (.71)	1.89 (0.63)	2.10 (0.65)	<0.001	0.036
Depression (1–24)	6.72 (6.02)	5.83 (5.31)	8.50 (6.04)	<0.001	<0.001

The crude results from multinomial logistic regressions (Table 3) showed that physicians and nurses were less likely to report nondrinking relative to drinking. Comparing nondrinking to drinking, males, those with a partner, depression, and burnout were more likely to report drinking, while HWCs with children living at home were more likely to report nondrinking. Further, HCWs with 5–15 years of work experience were more likely to report nondrinking compared to those with >15 years of work experience. Men, relative to women, were more likely to report problem drinking than drinking. HCWs with scores indicating depression or burnout were less likely to report problem drinking than drinking. No other significant relative differences were observed.

**Table 3:** Multinominal logistic regression for nondrinking, drinking, and problem drinking

		**Univariate model**				**Model 1** ^a^				**Model 2** ^a^				**Model 3** ^a^			
		**95% CI**				**95% CI**				**95% CI**				**95% CI**			
		**OR**	**Lower**	**Upper**	**Sig.**	**OR**	**Lower**	**Upper**	**Sig.**	**OR**	**Lower**	**Upper**	**Sig.**	**OR**	**Lower**	**Upper**	**Sig.**
Nondrinking^b^	Profession																
	Physicians	0.46	0.40	0.53	<0.001	0.44	0.38	0.52	<0.001	0.46	0.38	0.54	<0.001	0.45	0.38	0.54	<0.001
	Nurses	0.54	0.47	0.62	<0.001	0.53	0.46	0.61	<0.001	0.54	0.47	0.63	<0.001	0.54	0.46	0.63	<0.001
	Nurse assistants																
	Male (ref female)	0.74	0.64	0.86	<0.001	0.86	0.73	1.02	0.083	0.84	0.71	1.00	0.050	0.85	0.71	1.03	0.092
	Age	1.00	0.99	1.00	0.240	1.01	1.00	1.02	0.106	1.01	1.00	1.02	0.111	1.01	1.00	1.02	0.042
	Years of working experience																
	<5	1.10	0.93	1.30	0.288	1.56	1.22	2.00	<0.001	1.48	1.15	1.90	0.002	1.53	1.17	2.00	0.002
	5–10	1.37	1.18	1.61	<0.001	1.52	1.24	1.87	<0.001	1.44	1.17	1.78	<0.001	1.42	1.14	1.78	0.002
	10–15	1.46	1.25	1.71	<0.001	1.55	1.29	1.87	<0.001	1.52	1.26	1.84	<0.001	1.58	1.29	1.94	<0.001
	>15																
	Partner (ref no partner)	0.63	0.55	0.73	<0.001	0.61	0.52	0.72	<0.001	0.61	0.52	0.72	<0.001	0.59	0.50	0.70	<0.001
	Children living at home (no children)	1.27	1.13	1.43	<0.001	1.53	1.33	1.75	<0.001	1.51	1.32	1.73	<0.001	1.43	1.23	1.65	<0.001
																	
	Burnout	1.83	1.49	2.25	<0.001					1.71	1.38	2.12	<0.001				
	Depression	1.75	1.41	2.18	<0.001									1.69	1.34	2.11	<0.001
Problem drinking^b^	Profession																
	Physicians	0.95	0.69	1.31	0.754	0.78	0.54	1.13	0.186	0.81	0.56	1.18	0.278	0.74	0.51	1.08	0.123
	Nurses	1.07	0.79	1.47	0.658	1.06	0.77	1.46	0.837	1.09	0.78	1.52	0.617	1.02	0.73	1.43	0.913
	Nurse assistants																
	Male (ref female)	1.36	1.03	1.79	0.028	1.52	1.12	2.07	0.007	1.54	1.13	2.11	0.006	1.61	1.17	2.22	0.004
	Age	1.01	0.99	1.02	0.275	1.02	1.00	1.03	0.088	1.02	1.00	1.03	0.078				
	Years of working experience																
	<5	0.91	0.63	1.31	0.605	1.31	0.78	2.21	0.312	1.32	0.78	2.23	0.310	1.37	0.80	2.33	0.251
	5–10	0.92	0.65	1.31	0.651	1.07	0.70	1.74	0.660	1.04	0.65	1.65	0.880	1.13	0.71	1.79	0.616
	10–15	1.19	0.86	1.65	0.309	1.35	0.92	1.98	0.131	1.33	0.90	1.97	0.151	1.44	0.97	2.13	0.072
	>15																
	Partner (ref no partner)	0.89	0.64	1.23	0.474	0.84	0.59	1.18	0.307	0.79	0.56	1.12	0.191	0.85	0.60	1.21	0.370
	Children living at home (no children)	1.20	0.93	1.53	0.158	1.32	1.00	1.74	0.054	1.34	1.01	1.78	0.043	1.31	0.99	1.75	0.064
	Burnout	1.58	1.03	2.44	0.039					1.55	0.98	2.44	0.060				
	Depression	2.57	1.78	3.71	<0.001									2.79	1.91	4.07	<0.001
	−2 Log Likelihood					4137.197 (*P* < .001)				4406.890 (*P* < .001)				4191.414 (*P* < .001)			

^a^Models adjusted with all presented variables.

^b^Drinking was the base category

Few changes to the crude results are observed when all demographic variables are jointly added (Model 1). The exceptions are that there is no longer a statistical difference between men’s and women’s likelihood to report nondrinking relative to drinking (*P* = .083). Lastly, in Models 2 and 3, when burnout and depression were additionally added, both the significance level and OR remained at the same level as in Model 1. One exception was that males were again statistically significantly less likely to report nondrinking relative to drinking when adjusting for burnout and depression, respectively.

Focusing on comorbidity, HCWs reporting burnout, compared to those without burnout, were 70% more likely also to report nondrinking (OR: 1.71, 95% CI: 1.38–2.12) relative to drinking. Meanwhile, no differences in burnout were observed between drinking and problem drinking. Similarly, HCWs reporting depression, compared to those without depression, were more likely to report nondrinking relative to drinking (OR: 1.69, 95% CI: 1.34–2.11) and problem drinking relative to drinking (OR: 2.79, 95% CI: 1.91–4.07).

Sensitivity analysis with nondrinking as the base category showed no significant differences between HCWs reporting burnout relative to no burnout (OR: .85, 95% CI: .53–1.37, *P* = .513). On the other hand, HCWs reporting depression, compared to those who did not, were more likely also to report problem drinking relative to nondrinking (OR: 1.62, 95% CI: 1.08–2.42, *P* = .020).

Lastly, the stratified analyses (Table 4) indicated some demographic variations between the three professions in the levels of use of alcohol. With regard to the differences between professions in the levels of use of alcohol, statistically significant differences between nondrinking and drinking were observed for years of working experience for physicians and nurse assistants but not for nurses and having a partner and children living at home for nurses and nurse assistants but not for physicians. While sex differences in drinking patterns were observed for nurses and nurse assistants, no such differences were observed among physicians. Comorbidity between depression and problem drinking was observed across all professions (physicians OR: 3.52 95% CI: 1.86–6.63; nurses OR: 2.02 95% CI: 1.06–3.84; nurse assistants OR: 2.96 95% CI: 1.44–6.08).

**Table 4:** Stratified multinomial logistic regression analysis for physicians, nurses, and nurse assistants for nondrinking, drinking, and problem drinking. Adjusted models

		**Physicians**				**Nurses**				**Nurse assistants**			
**Burnout**													
		**95% CI**				**95% CI**				**95% CI**			
		**OR**	**Lower**	**Upper**	**Sig.**	**OR**	**Lower**	**Upper**	**Sig.**	**OR**	**Lower**	**Upper**	**Sig.**
Nondrinking^a^	Male (ref female)	0.82	0.65	1.03	0.091	0.68	0.47	0.99	0.042	1.10	0.75	1.64	0.605
	Age	1.02	1.01	1.04	0.014	1.00	0.99	1.02	0.884	1.01	0.99	1.02	0.375
	Years of working experience												
	<5	1.75	1.03	2.97	0.038	1.26	0.82	1.94	0.288	1.71	1.09	2.69	0.020
	5–10	1.76	1.14	2.73	0.012	1.36	0.93	2.00	0.112	1.45	1.05	2.02	0.026
	10–15	1.65	1.13	2.42	0.010	1.31	0.94	1.81	0.108	1.82	1.33	2.49	<0.001
	>15												
	Partner (ref no partner)	0.51	0.37	0.70	<0.001	0.61	0.47	0.80	<0.001	0.72	0.55	0.93	0.011
	Children living at home (ref no children)	1.19	0.92	1.54	0.186	1.47	1.17	1.84	<0.001	1.86	1.46	2.36	<0.001
	Burnout	1.78	1.23	2.56	0.002	1.60	1.08	2.35	0.019	1.68	1.17	2.40	0.005
Problem drinking^a^	Male (ref female)	1.21	0.79	1.86	0.387	1.74	1.03	3.00	0.040	2.23	1.05	4.74	0.037
	Age	1.04	1.01	1.08	0.013	1.02	0.99	1.05	0.211	0.99	0.96	1.02	0.438
	Years of working experience												
	<5	1.82	0.67	4.92	0.240	1.62	0.74	3.52	0.225	0.66	0.17	2.49	0.536
	5–10	1.35	0.57	3.19	0.496	0.68	0.29	1.60	0.379	1.50	0.71	3.18	0.286
	10–15	1.85	0.93	3.67	0.078	1.33	0.72	2.44	0.361	0.92	0.38	2.22	0.850
	>15												
	Partner (ref no partner)	0.92	0.46	1.86	0.820	0.84	0.49	1.46	0.541	0.71	0.38	1.31	0.268
	Children living at home (ref no children)	1.16	0.71	1.89	0.55	1.54	1.00	2.39	0.050	1.11	0.61	2.01	0.736
	Burnout	1.08	0.46	2.52	0.865	1.64	0.77	3.53	0.201	1.90	0.86	4.19	0.114
	−2 Log likelihood	947.942 (*P* < .001)				1079.050 (*P* < .001)				937.116 (*P* < .001)			
**Depression**													
		**95% CI**				**95% CI**				**95% CI**			
		**OR**	**Lower**	**Upper**	**Sig.**	**OR**	**Lower**	**Upper**	**Sig.**	**OR**	**Lower**	**Upper**	**Sig.**
Nondrinking^a^	Male (ref female)	0.87	0.68	1.12	0.281	0.69	0.46	1.04	0.074	0.99	0.64	1.53	0.950
	Age	1.02	1.00	1.04	0.021	1.01	0.99	1.02	0.521	1.01	0.99	1.02	0.323
	Years of working experience												
	<5	1.94	1.10	3.42	0.021	1.22	0.78	1.92	0.388	1.98	1.23	3.17	0.005
	5–10	1.84	1.15	2.95	0.011	1.38	0.91	2.03	0.138	1.37	0.97	1.94	0.079
	10–15	1.87	1.25	2.80	0.002	1.30	0.92	1.84	0.138	1.88	1.34	2.64	<0.001
	>15												
	Partner (ref no partner)	0.49	0.35	0.68	<0.001	0.61	0.46	0.81	<0.001	0.68	0.52	0.88	0.004
	Children living at home (no children)	1.20	0.91	1.59	0.195	1.34	1.06	1.70	0.016	1.74	1.35	2.24	<0.001
	Depression	1.81	1.18	2.77	0.007	1.65	1.15	2.38	0.007	1.57	1.06	2.35	0.026
													
Problem drinking^a^	Male (ref female)	1.30	0.84	2.02	0.237	1.73	0.99	3.03	0.056	2.47	1.15	5.30	0.021
	Age	1.04	1.01	1.08	0.020	1.03	1.00	1.06	0.059	1.00	0.97	1.03	0.918
	Years of working experience												
	<5	1.76	0.62	5.00	0.286	1.74	0.80	3.79	0.162	0.72	0.19	2.77	0.636
	5–10	1.52	0.63	3.64	0.352	0.72	0.31	1.71	0.459	1.47	0.70	3.10	0.309
	10–15	1.97	0.97	3.99	0.059	1.42	0.77	2.61	0.262	0.97	0.40	2.35	0.945
	>15												
	Partner (ref no partner)	1.11	0.53	2.32	0.791	0.84	0.48	1.46	0.530	0.81	0.40	1.45	0.491
	Children living at home (no children)	1.12	0.67	1.85	0.674	1.57	1.01	2.44	0.047	1.10	0.61	1.98	0.760
	Depression	3.52	1.86	6.63	<0.001	2.02	1.06	3.84	0.032	2.96	1.44	6.08	0.003
	−2 Log likelihood	896.051 (*P* < .001)				1516.582 (*P* < .001)				1286.547 (*P* < .001)			

^a^Drinking was the base category.

## Discussion

This cross-sectional study used a large representative sample of Swedish HCWs (physicians, nurses, and nurse assistants) to investigate prevalences of nondrinking, drinking, and problem drinking, as well as potential comorbidity between the use of alcohol and burnout and depression, respectively. The prevalence of problem drinking among Swedish HCWs was 3.7%, with variations across subgroups of HCW. A fifth of the HCWs reported not drinking alcohol. Also, we found comorbidity between problem drinking and depression but not between problem drinking and burnout. In this discussion, we will focus on the results concerning drinking problems.

The prevalence of problem drinking found in this study is somewhat higher than for HCWs in other countries (Foli *et al*. 2021; Thiebaud *et al*. 2021; Wijeratne *et al*. 2021; Mo *et al*. 2022) but lower than the general working population in Sweden and workers in the Swedish health and social care sector (Tareq *et al*. 2024). While demographic differences in prevalence were observed for those who did not drink alcohol, only sex differences were observed for those with problem drinking, with males at high risk of reporting problem drinking. The study results contrast a recent systematic review, which showed that sex did not predict hazardous alcohol use in HCWs (Halsall *et al*. 2023) but aligns with another Swedish study showing that men report higher prevalences of problem drinking (Tareq *et al*. 2024). Further, our results indicated that sex differences occurred only among nurses and nurse assistants, two female-dominated occupations, and not for physicians with a balanced sex distribution. The nonsignificant sex differences for physicians do not align with previous results reporting that the harmful use of alcohol is more common in male physicians compared to females (Rosta and Aasland 2013; Mo *et al*. 2022; Tao *et al*. 2023). Differences in the prevalence of various aspects of harmful alcohol use across studies may be due to country contexts but also different measurements have been used (Waithera *et al*. 2024).

Although the highest mean level of burnout and depression was found among those HCWs reporting problem drinking, comorbidity seemed to only exist between depression and problem drinking when adjusting for other variables. Our results differ from a previous review (Rosta and Aasland 2013) but align with a study showing that physicians with depressive symptoms were more likely to report alcohol dependence (Oreskovich *et al*. 2015). Furthermore, both burnout and depression, and especially burnout, were more common among HCWs who reported not drinking alcohol. Taken together, more longitudinal research is needed to disentangle how burnout and depression, respectively, are associated with the use of alcohol.

Our results should be viewed against the backdrop that they were collected in 2022, shortly after the COVID-19 pandemic. Research indicates that the prevalence of harmful alcohol use has increased during the pandemic (Beiter *et al*. 2022). Also, the COVID-19 pandemic exposed HCWs to high workloads and stress, with global evidence demonstrating an increase in alcohol consumption (Arble *et al*. 2023; Halsall *et al*. 2023). Although some HCWs may have increased their alcohol drinking during this period in a responsible manner, the combination of increased emotional distress and substance use is potentially critical (Choflet *et al*. 2021) and fatal for frontline healthcare professionals (Gossop 2001) and needs sudden attention.

Problem drinking among HCWs is of concern as it can impair their health, performance, professional conduct, cognitive function, and decision-making, potentially leading to medical errors and poor patient safety (Kilian *et al*. 2021; Mc Magh *et al*. 2023). While we recommend further longitudinal studies confirming the associations between problem drinking among HCWs and quality of care, actions are needed to reduce problem drinking and mental ill health in HCWs.

### Strengths and limitations

The strengths of the study are the use of validated tools to assess problem drinking, burnout, and depression and the large sample size, representing the Swedish population of HCW studied. However, the study has some limitations that should be considered when interpreting the results. First, the outcomes are based on self-reports of our study variables (alcohol use, burnout, and depression). Recall bias could occur, and underreporting may be more common than overreporting in samples of HCWs. The stigma associated with drinking problems (Hyman *et al*. 2017), social desirability and underestimation of alcohol use might impact the reporting of alcohol use (McCusker et al. 2002). Second, this study was based on cross-sectional survey data, which cannot infer causal relationships between exposure and outcome variables.

Lastly, there exist several instruments that measure alcohol use in different ways. We chose the CAGE because we wanted a short, feasible, and validated instrument to measure alcohol use as a drinking problem (Dhalla and Kopec 2007). The modified version of CAGE has been used in other Swedish Studies and is therefore valuable to use for comparability (Tareq *et al*. 2024). The CAGE instrument has received critique as it includes lifetime use of alcohol (Dhalla and Kopec 2007). In the LOHHCS survey, respondents are first asked to indicate how often they drank alcohol in the last 12 months. Those reporting that they had been drinking for the last 12 months were asked to answer the CAGE question, limiting the risk that the problem of drinking occurred years ago. Moreover, although a CAGE score of ≥2 is conventionally used to identify alcohol-related issues, some researchers have suggested employing a score of ≥1 for general screening purposes, arguing that this would increase the tool’s sensitivity and enhance the detection of early-stage cases (Castells and Furlanetto 2005). However, a meta-analysis of CAGE studies concluded that a cut-off of 1 significantly diminishes the tool’s specificity, thereby increasing the risk of false positives (Aertgeerts *et al*. 2004). We, therefore, used ≥2 as the cut-off.

## Conclusions

This study demonstrated that ~3.7% of Swedish HCWs reported problem drinking and that those also had a higher likelihood of reporting depression but not burnout. Male HCWs were more likely than females to report problem drinking. Although not higher than in the general population, it is imperative to prevent problem drinking among HCWs as it may cause great personal suffering and poor patient safety.

## Data Availability

The datasets analysed during the current study are not publicly available due to ethical regulations, but aggregated data are available from the corresponding author upon reasonable request.
